# Internet-based stress recovery intervention for adolescents: study protocol for a randomized controlled trial

**DOI:** 10.1186/s13063-023-07188-1

**Published:** 2023-03-08

**Authors:** Paulina Zelviene, Agniete Kairyte, Austeja Dumarkaite, Augustė Nomeikaite, Evaldas Kazlauskas

**Affiliations:** Center for Psychotraumatology, Institute of Psychology, Vilnius University, M. K. Ciurlionio str. 29, Vilnius, Lithuania

**Keywords:** Stress, Stress recovery, Internet intervention, Adolescents

## Abstract

**Background:**

Research reveals a high prevalence of stressors in adolescence. Mental health in adolescence is highly related to life-stressors exposure and difficulties in adjusting to stressors. Therefore, interventions for recovery from stress are in high demand. The study aims to evaluate the efficacy of the Internet-based stress recovery intervention for adolescents.

**Methods:**

A two-arm randomized controlled trial (RCT) on the efficacy of the FOREST-A—an Internet-based stress recovery intervention for adolescents—will be conducted. The FOREST-A is an adapted version of stress recovery intervention initially developed for healthcare workers. FOREST-A is a third-wave cognitive behavioral therapy and mindfulness-based Internet-delivered 4-week psychosocial intervention, which comprises six modules: Introduction, Relaxation, Psychological detachment, Mastery, Control, and Summary. The intervention will be evaluated using the two-arm RCT with intervention and care as usual (CAU) condition at pre-test, post-test, and 3-month follow-up. The measured outcomes will be stress recovery, adjustment disorder, generalized anxiety and depression symptoms, psychological well-being, and perceived positive social support.

**Discussion:**

The study will contribute to the development of Internet interventions—easily and broadly accessible tools—for the enhancement of adolescents’ stress recovery skills. Based on the study’s findings, further development of the FOREST-A, including upscaling and implementation, is foreseen.

**Trial registration:**

ClinicalTrials.gov NCT05688254. Registered on January 6, 2023.

## Administrative information

**Table:** 

Title {1}	Internet-based stress recovery intervention for adolescents: study protocol for a randomized controlled trial
Trial registration {2a and 2b}.	The planned study is a psychosocial intervention. See item #2a, the Trial was registered on ClinicalTrials.gov (https://clinicaltrials.gov/ct2/show/NCT05688254?term=FOREST-A&draw=2&rank=1) ClinicalTrials.gov NCT05688254. Registered on January 6, 2023.
Protocol version {3}	Version 2.0. 10 February 2023
Funding {4}	This project has received funding from European Social Fund (project No: 01.2.2-LMT-K-718-03-0072) under grant agreement with the Research Council of Lithuania (LMTLT).
Author details {5a}	Paulina Zelviene, Center for Psychotraumatology, Institute of Psychology, Vilnius University, M. K. Ciurlionio str. 29, Vilnius, Lithuania Agniete Kairyte, Center for Psychotraumatology, Institute of Psychology, Vilnius University, M. K. Ciurlionio str. 29, Vilnius, Lithuania Austeja Dumarkaite, Center for Psychotraumatology, Institute of Psychology, Vilnius University, M. K. Ciurlionio str. 29, Vilnius, Lithuania Augustė Nomeikaite, Center for Psychotraumatology, Institute of Psychology, Vilnius University, M. K. Ciurlionio str. 29, Vilnius, Lithuania Evaldas Kazlauskas, Center for Psychotraumatology, Institute of Psychology, Vilnius University, M. K. Ciurlionio str. 29, Vilnius, Lithuania
Name and contact information for the trial sponsor {5b}	The information about the sponsor of the study Vilnius University 3 Universiteto St., LT-01513 Vilnius Phone: +370 5 268 7000 E-mail:infor@cr.vu.lt Institutional Code: 211950810 VAT Number: LT119508113
Role of sponsor {5c}	The sponsor played no part in study design; collection, management, analysis, and interpretation of data; writing of the report; and the decision to submit the report for publication.

## Background

Adolescence is a developmental stage marked by rapid changes and adjustment challenges to these changes. Studies show that exposure to life stressors in adolescence is highly prevalent. Findings from recent studies reveal the experience of as high as 76% of recent significant stressors (e.g., difficulties at school, illness of family member, etc.) over the last year in adolescence in European or Asian countries. Moreover, around half of adolescents experience multiple stressors [1]. Furthermore, these stressors often are associated with difficulties in school (39.0%), difficulties in peer relationships (28.6%), or family conflicts (23.3%). School and achievement-related stressors are prevalent from early adolescence, and the level of stress in this domain has a tendency to increase from early to mid-adolescence [2].

Stressor exposure is associated with diverse negative consequences for mental health. Difficulties of coping with life stressors often may lead to adjustment disorder in adolescence [1]. Lack of stress management and recovery skills could be associated with poor school performance and increased depressive symptoms [3]. Youths who reported more daily stressors also experience more physical health symptoms and negative affect [4]. In order to prevent adverse outcomes of stressor exposure on mental health, it is essential to promote stress recovery skills in adolescents [5, 6].

Internet-based interventions for mental health promotion are among the most promising solutions, which received considerable interest from practitioners and scientists recently [7, 8]. In general, studies find Internet-based interventions to be effective, but further development in the field is needed [7, 9]. While most studies in the Internet-based interventions field have been conducted in adult samples, there is an increasing number of Internet-based psychosocial interventions for various mental health disorders and conditions in adolescence. Most commonly, Internet-based intervention trials target depression [10], anxiety [11], or chronic medical conditions [12].

Internet-based stress recovery intervention For Recovery from Stress—Adolescent Version (FOREST-A) was created to address the need to develop and enhance stress recovery skills in adolescents exposed to life stressors and experiencing high levels of stress. The intervention aims to prevent mental health problems in stressor-exposed adolescents. The FOREST-A intervention is grounded on a mindfulness-based approach, which has been recognized as improving the self-regulation of emotions, behavior, and cognitive processes. These skills contribute to coping with stress [13]. FOREST-A was developed as a modification of the FOREST intervention, initially created as a stress recovery intervention for healthcare workers [14]. Findings from the RCT study revealed high effect sizes of the FOREST intervention [15]. The FOREST-A program and design of the study were adapted to the needs of adolescents. At the same time, the primary approach and structure of the modules remain similar to the original FOREST intervention.

The main objective of the study is to evaluate the efficacy of the Internet-based stress recovery intervention for adolescents (FOREST-A) in a two-arm RCT by comparing outcomes of the stress-related psychological indicators in the intervention and care as usual (CAU) groups. The secondary objectives of the study are to evaluate the effects of the FOREST-A: (1) on stress recovery skills, (2) on adjustment disorder symptoms, (3) on anxiety symptoms, (4) on general health and well-being, (5) on perceived social support, and (6) to evaluate the FOREST-A itself, how user-friendly and acceptable for study participants it is.

## Methods

### Study design and setting

A two-arm randomized controlled trial (RCT) of the efficacy of an Internet-based stress recovery intervention for adolescents is planned. Participants will be adolescents aged 15–19 years, studying in high school and who have indicated exposure to daily life stressors, such as school or interpersonal stressors. In order to achieve the objectives of the study, the planned sample size is 300 adolescents from Lithuania. To assess the efficacy of the 6-module four-week psychosocial intervention, the evaluation in the intervention and care as usual (CAU) groups will be carried out at the three-time points: (1) pre-intervention, (2) post-intervention, and (3) at the 3-month follow-up. These measurements will assess adolescents’ stress recovery experiences, adjustment, perceived stress, depression, anxiety, psychological well-being, and positive social support. The demographic data questionnaire will be presented at the pre-intervention test, while questions on the evaluation of the program will be asked in the follow-up. Implementing the intervention will not require alteration to usual care pathways (including the use of any medication), and these will continue for both trial arms. There are no restrictions regarding concomitant care during the trial. Both study groups will be provided with the information about psychological support options at their school. All participants of this study will have the same access to mental health care as all adolescents in Lithuania. Details of study enrolment, intervention, and assessments are presented in Table 1.

**Table 1:** Enrollment, interventions, and assessments of the FOREST-A

	Enrollment	Allocation	Post-allocation	
Time point	*t*_*1*_		*t*_*2*_	*t*_*3*_
Enrollment				
Informed consent	X			
Informed parent consent	X			
Assessment	X			
Eligibility screen		X		
Randomization		X		
Final allocation		X		
Access to intervention				
Intervention group		X		
Care as usual group				X
Assessments				
Recovery experiences	X		X	X
Adjustment	X		X	X
Stress	X		X	X
Depression	X		X	X
Anxiety	X		X	X
Psychological well-being	X		X	X
Perceived positive social support	X		X	X

Short telephone interviews or text messages will be used to enhance participants’ engagement with the program [16]. Participants will be contacted by the administrator on three occasions: (1) before the program, (2) during the participation in the program (second week), and (3) after the program. During the first and second calls, the study administrators will answer participants’ questions about the technical aspects of using the program. During the third call, the study administrators will ask participants to share their general impressions of using the program and their recommendations on how to make it more user-friendly.

The study was approved by the Vilnius University Committee on Research Ethics in Psychology (December 12, 2022, Registration No. (1.5 E) 250000-S-646). Minor-aged participants (< 18 years) will be provided with informed consent for their parents/legal guardians containing information about the study, intervention, and confidentiality of the data. After informed consent from the parent/guardian is obtained, the study team will proceed with enrolment in the study and program. The enrolment form will notify the adolescent of the informed parental consent. Participants will also be informed that no information will be shared with parents about what the participant completes in the program and answers to the survey questions, except in the case of perceived threats to the health or life of the participant or others. Participants will also be informed that the participation is voluntary and they can withdraw from the intervention at any time and all their provided data can be removed upon their request. Participants will be informed that the data collected during the study will only be presented in a generalized form in scientific presentations and papers, without the possibility of identifying a specific individual. This trial does not involve collecting biological specimens for storage. Additional consent to participate in the study will be obtained from adolescents. Participation in the program for minor-aged participants (< 18 years) would be available only if they provided parental consent before the planned launch of the intervention (the time specified in the registration form). No parental consent will be needed for adolescents aged 18 years or older, and therefore, they can be enrolled in the study after providing informed consent themselves.

**Fig. 1:**
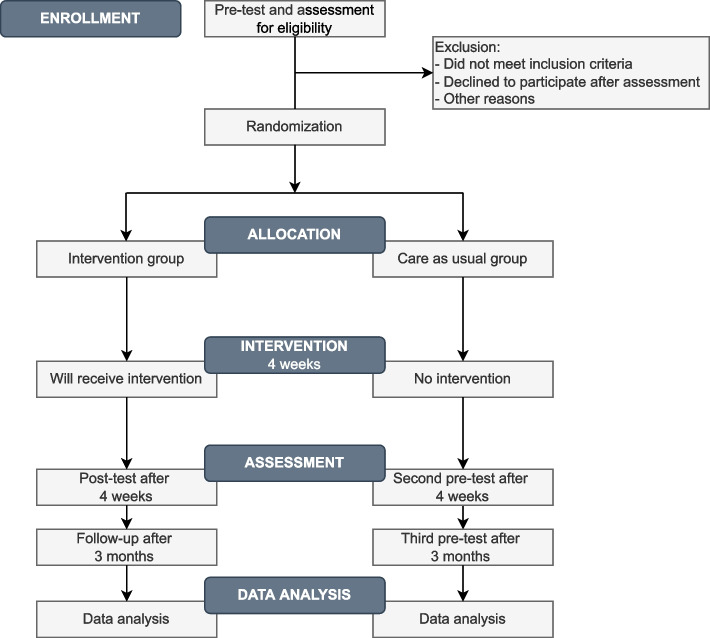
Flowchart of the FOREST-A intervention

All participants who provided informed consent will be asked to complete the measures in a secure online platform designated for the research survey. Eligibility for inclusion in the study will be assessed based on the responses from participants at the pre-intervention assessment. Those who do not meet the eligibility criteria will be informed of this and given information on other options for psychosocial services in the school or community (Fig. 1).

### Participants and inclusion/exclusion criteria

The information about the intervention would be sent to various high school institutions across Lithuania. The invitations to participate in the study and intervention would be spread in collaboration with contact persons in schools.

The sample will comprise self-referred adolescents who experience high levels of stress in daily life. The inclusion criteria for the participation: (1) adolescents aged 15–19, (2) studying at the high school in Lithuania; (3) comprehending Lithuanian, (4) parental and own consent for minor-aged participants (< 18 years) or only own consent for adolescents aged 18 years or older to participate in the study provided, (5) have access to a device (such as a tablet, phone or computer) with an Internet connection. Exclusion criteria: (1) acute psychiatric condition/crisis, (2) no recent life-stressor exposure, or low levels of stress.

### Randomization

Participants will be randomly allocated to either the intervention group or the care as usual group (1:1 allocation ratio). Randomization will be carried out by an independent researcher using the random number calculation procedure at www.random.org. Adolescents in the intervention group will receive access to the FOREST-A intervention immediately after allocation, while those in the care as usual group condition will have the possibility to access social or mental health services in the community.

### Intervention condition

FOREST-A is an online psychosocial intervention program for adolescents aimed at enhancing stress recovery skills. FOREST-A was adapted from the stress recovery program developed specifically for healthcare staff (details of the program and description of content can be found in the study protocol by Jovarauskaite et al., 2021). The program was found to be effective in improving stress recovery skills of psychological detachment (*d* = 0.83), relaxation (*d* = 0.93), mastery (*d* = 0.64), and control (*d* = 0.46) at post-treatment, and all, except control, were improved at the 3-month follow-up. Users also reported positive feedback on good usability of the intervention [15]. The program is based on cognitive behavior therapy (CBT), including the third-wave CBT [17] mindfulness—approach. It is an online intervention that can be used via a computer, tablet, or smartphone. The program is comprised of six main modules:

1. Introduction. The first session provides information about the program and its usage, psychoeducation and exercises related to stress, burnout, and four recovery skills (relaxation, psychological detachment, mastery, and control).
2. Relaxation. In order to enhance participants’ relaxation skills, the second session provides psychoeducation and exercises related to physical sensations, body relaxation, and sleep.
3. Psychological detachment. The third session provides opportunity to enhance participants’ psychological detachment skills. It includes psychoeducation and exercises related to psychological detachment (both physical and mental) and intrusive thoughts.
4. Mastery. The fourth session provides psychoeducation and exercises related to mastery of daily life (other than school-related) as well as physical activity. These activities include enjoyable tasks that require some competence (e.g., reading, cooking, and doing sports, among others). The participant chooses what is relevant for him/her and what can bring the feeling of being competent. Its main aim is to enhance participants’ mastery skills in individually chosen activity or activities.
5. Control. The fifth session provides psychoeducation and exercises related to control over one’s life and the day’s structure. By reflecting on daily goals and changing unnecessary and bothering activities to more pleasant and relaxing ones, the participant is encouraged to take responsibility for his/her well-being. Its main aim is to enhance participants’ control skills.
6. Summary and keeping the change alive. The last session summarizes the program with its main aspects, psychoeducation, and exercises related to consolidation of new skills. Its main aim is to enhance the integration of new skills into participants’ lives.

All the sessions are similar in structure: they include psychoeducation (written texts and video recordings), several exercises (written tasks and audio recordings — mindfulness-based activities), and the possibility of contacting a psychologist. FOREST-A maintains the same theoretical background, delivery form, and module structure as the previous stress recovery intervention FOREST designed for healthcare staff, which yielded positive outcomes. However, important adaptations were made to suit the adolescent developmental stage to make it relevant for adolescents. The adapted version is shorter in time — the program will last 4 weeks instead of 6 weeks. Access to a new session will be provided every 5 days over the 4 weeks, and once accessible, sessions will remain available throughout the remaining program. Also, and most importantly, the content and the language are adapted to fit the age and needs of adolescents. For example, the program is focused on studies — instead of work-related stressors.

The psychologists will not be providing feedback on all the completed modules in the intervention. However, psychologists will be available on demand and will respond if participants will need any support. Psychologists’ role will include answering participants’ messages within 24 h if they have questions related to the program and personal condition and want to share their experiences. Psychologists will be trained on how to deliver intervention and respond to participants’ requests according to the provided guidelines, and they will participate in weekly supervisions to discuss the cases. If needed, participants will be referred for specialized or acute mental health services. A team of clinical psychologists will be involved in the delivery of the FOREST-A program. The FOREST-A will be delivered through a secure online platform, which has been developed and designed specifically for delivering Internet-based psychosocial interventions.

### Data management

The storage and management of the data will be carried out according to the European and National institutional regulations. Secure online platform will be used for the delivery of Internet-intervention, communication with the specialist, and for collection of outcome data. Each participant will have their own account with personal ID and a password. Only researchers who are involved in the study will have access to the intervention and data via their personal ID and a password.

All participants will be informed about the confidentiality of the data and that the published results will be presented without the possibility of identifying a specific individual. The data will be exported from the platform only for the statistical analysis purposes and will be stored in the university’s electronic repository for the storage of such data. The personal information will be separated from the main data file and will be accessible only to the principal investigators of this study. Participants will be informed that they can withdraw from the intervention and the study whenever they wish, and their personal intervention account and data can be removed from the intervention and the study upon request.

### Primary outcome

The Recovery Experience Questionnaire (REQ) [18] will be used to measure stress recovery. The REQ was previously translated into Lithuanian and used in the adult sample in Lithuania [15]. The revised child and adolescent version of the REQ adapted for children and adolescents (REQ-CA) will be used in this study. The REQ-CA comprises 16 items and four subscales (with four items per each subscale) to measure recovery experience components: psychological detachment, mastery, relaxation, and control. Participants are asked to provide responses to each of the items on a 5-point Likert scale, ranging from *Totally disagree* (= 1) to *Totally agree* (= 5). The total score of the REQ-CA is a sum of all scale items and ranges from 16 to 80. The REQ-CA subscales scoring is a sum of items comprising the subscale with a range from 4 to 20. Higher scores on the REQ-CA and subscales indicate higher stress recovery skills. Previous studies showed good psychometric properties of the REQ in adult samples [15, 19].

### Secondary outcomes

The Adjustment Disorder New Module-8 Child and Adolescent Version (ADNM-8-CA) will be used to measure exposure to stressors in the past month and ICD-11 symptoms of adjustment disorder [1, 20]. The ADNM-8-CA stressors list includes 16 potentially stressful events relevant to adolescents (e.g. parents’ divorce, difficulties in school, the end of the friendship). Participants are asked to provide binary answers *No* or *Yes* if they have experienced any of the listed stressors over the last 12 months. The assessment of adjustment disorder symptoms includes eight symptom items. The ADNM-8-CA measures two ICD-11 adjustment disorder symptoms: (1) preoccupation with the stressor (4 items) and (2) failure to adapt (4 items). A 4-point Likert scale, ranging from *Never* (= 1) to *Often* (= 4) is used to evaluate the frequency of the listed symptoms. The total score of the ADNM-8-CA range from 1 to 32. A higher total score which is a sum of all the ADNM-8-CA symptom items, indicates higher adjustment problems, and a score ≥ 23 indicates probable adjustment disorder. The ADNM-8-CA was previously used in a large Lithuanian adolescent sample, and good psychometric properties have been reported [1].

The Generalized Anxiety Disorder-7 (GAD-7) [21] will be used to measure generalized anxiety symptoms that bothered participants during the past 2 weeks. The GAD-7 comprises 7 items with the possible answers ranging on a 4-point Likert scale from *Not at all* (= 0) to *Nearly every day* (= 3). The total score of the GAD-7 range from 0 to 21, with higher scores indicating higher generalized anxiety problems. The GAD-7 is applicable to adolescents from 12 years old. However, GAD-7 has not previously been used in a sample of Lithuanian adolescents. Nevertheless, the GAD-7 showed good psychometric properties in adult samples in Lithuania [22, 23].

The Patient Health Questionnaire-9 (PHQ-9) [24] will be used to measure depressive symptoms that bothered participants during the past 2 weeks. The PHQ-9 comprises nine items, with the answers ranging from *Not at all* (= 0) to *Nearly every day* (= 3) on a 4-point Likert scale. The total score of the PHQ-9 may range from 0 to 27, with higher scores indicating higher depressive symptoms. The PHQ-9 is suitable for adolescents from 13 years old. The PHQ-9 has not been used in a sample of Lithuanian adolescents previously. The PHQ-9, used in adult samples in Lithuania, showed good psychometric properties [22, 23].

The WHO-5 Well-being Index (WHO-5) [25] will be used to measure general psychological well-being. The WHO-5 comprises five items measuring how an individual felt over the past 2 weeks with a Likert scale ranging *At no time* (= 0) to *All of the time* (= 5). The total score of the WHO-5 is the sum of all items multiplied by 4, and may range from 0 to 100, with a bigger score indicating higher well-being. The WHO-5 is suitable for children aged 9 and above. In Lithuanian adult samples, the WHO-5 has good psychometric properties [22, 23].

The Perceived Positive Social Support Scale (PPSS) [26] is a revised version of the Crisis Support Scale [27] measuring social support. The PPSS comprises four items, covering how the participant feels with family and friends (e.g., how often someone tends to listen when the participant wants to talk). Participants are asked to provide a response to each item on an 8-point Likert scale ranging from *Never* (= 0) to *Always* (= 7). The total score of PPSS may range from 0 to 28, with higher scores indicating higher perceived positive social support. The PPSS has been used previously in the adolescent sample in Lithuania and showed good psychometric properties [26].

### Other measures

After the intervention (at post-intervention), the program evaluation questions will be given. The questions will cover features of usage of the program (e.g., what device the participant used most often; how often during the week participant used the program). Also, questions will include an evaluation of the general satisfaction and usefulness of the program, subjective psychological and physical well-being changes while using the program, and whether the participant will recommend the program to other adolescents.

### Statistical analyses

Repeated measures ANOVA will be applied to evaluate group × time effects of the intervention. Within-group and between-group effect sizes will be used to estimate the role of intervention on the change of the primary and secondary outcomes.

### Composition of the coordinating center and trial steering committee

The Vilnius University Center for Psychotraumatology located at the Institute of Psychology will be responsible for the coordination of the study. The steering committee members will be Ph.D. level researchers and Ph.D. students who also are experienced clinical psychologists. The principal investigator will be responsible for the delivery of the study, coordination and supervision of the team work, and communication with the study sponsor. Program design team will be responsible for the content and overall design of the intervention. The trial manager will be responsible for the implementation of the intervention, collaboration with institutions and participants, and for day-to-day support for the trial, reviewing the participant reports about their psychological well-being during the intervention and contacting them for ensuring safety (e.g., reported suicidal ideation during the trial). The data manager will be responsible for data collection, protection, coding and analysis. The team will have a kick off meeting, regular meetings (at least once a week) for management and supervision and on-demand meetings.

The steering committee will have the weekly meetings for the auditing of study process and team work. The closing meeting will be set up after the trial is finished. All study management will be according to the trial application approved by the Vilnius University Committee on Research Ethics in Psychology.

## Discussion

Research on Internet-based interventions provides promising results and new trends in improving access to mental health services [28], while at the same time, the evidence for the outcomes of Internet-based interventions for youth is very limited [29–31]. This RCT aims to evaluate the efficacy of the Internet-based stress recovery intervention for adolescents (FOREST-A). The idea to create this intervention and plan a study came from the need to find efficient ways to help adolescents to cope with mental health challenges. Promising results on the effectiveness [15] of the Internet-based stress recovery intervention (FOREST) have been reported previously. On the other hand, FOREST was created for adult healthcare staff. Therefore, the modified version of FOREST-A might have different outcomes in the adolescent sample. If the FOREST-A is proven effective, this will lead to improvement in adolescents’ stress recovery skills and psychological well-being.

Despite the prediction of the effectiveness of the FOREST-A, the current study may have few limitations that will require attention while interpreting the results. These limitations arise due to the study design and the available resources. First, the program will be available to those adolescents who have an access to device with Internet connection and obtained an informed consent from their parents. It might be difficult to manage dropout rates, since this field of research is still developing and there are a lot of unknown factors related to this [32]. Also, there is a self-selection bias, since adolescents will be invited and not randomly selected to participate in the trial.

The study will also provide empirical knowledge about FOREST-A’s effects on adjustment disorder and anxiety symptoms, general health and well-being, and perceived social support. The study will also give knowledge about the program-related user experience factors (e.g., design, ease of use, engagement, and the possibility of communicating with the specialist).

## Trial status

The estimated start of the recruitment of participants is on March 1, 2023. The estimated finish of the study is on December 12, 2026. The trial was registered on January 6, 2023 via www.clinicaltrials.gov, No. NCT05688254.

## Data Availability

The data file will not be publicly available. Principal investigators will have access to the final dataset. An anonymous copy of the data file and statistical code of this study will be available from the corresponding author upon reasonable request, as is the full protocol.

## Abbreviations

FOREST-A: For Recovery from Stress—Adolescent Version
REQ-CA: Recovery Experience Questionnaire—Child and Adolescent Version
ADNM-8-CA: Adjustment Disorder New Module-8 Child and Adolescent Version
GAD-7: Generalized Anxiety Disorder-7
PHQ-9: Patient Health Questionnaire-9
WHO-5: The World Health Organization-5 Well-being Index
PPSS: Positive perceived social support
