# Correction: Internet-based stress recovery intervention for adolescents: study protocol for a randomized controlled trial

**DOI:** 10.1186/s13063-023-07439-1

**Published:** 2023-06-13

**Authors:** Paulina Zelviene, Agniete Kairyte, Austeja Dumarkaite, Augustė Nomeikaite, Evaldas Kazlauskas

**Affiliations:** Center for Psychotraumatology, Institute of Psychology, Vilnius University, M. K. Ciurlionio Str. 29, Vilnius, Lithuania

**Correction: Trials 24, 174 (2023)**

**https://doi.org/10.1186/s13063-023-07188-1**

The original publication of this article [1] contained an incorrect funding section. The incorrect and correct information is listed in this correction article, the original article has been updated.

Incorrect

This project has received funding from European Social Fund (project No: 01.2.2-LMT-K-718-03-0072) under grant agreement with the Research Council of Lithuania (LMTLT).

Correct

The project has received funding from European Regional Development Fund (project No: 01.2.2-LMT-K-718-03-0072) under a grant agreement with the Research Council of Lithuania (LMTLT)
